# Supplementary material for: Sculpting ultrastrong light–matter coupling through spatial matter structuring
Source: Nanophotonics. 2024 Jan 11;13(10):1909–15. doi: 10.1515/nanoph-2023-0604 (PMC11052535; doi:10.1515/nanoph-2023-0604)
Supplement: Supplementary file 1 — Supplementary Material Details [file j_nanoph-2023-0604_suppl_001.pdf]

Sculpting ultrastrong light-matter coupling through spatial matter structuring
Supplementary Material

Joshua Mornhinweg[1,4,*], Laura Diebel[1], Maike Halbhuber[1], Josef Riepl[1], Erika Cortese[2], Simone De Liberato[2,3], Dominique Bougeard[1], Rupert Huber[1], Christoph Lange[4,*]

[1]*Department of Physics, University of Regensburg, 93040 Regensburg, Germany*

[2]*School of Physics and Astronomy, University of Southampton, Southampton, SO17 1BJ, United Kingdom*

[3]*IFN - Istituto di Fotonica e Nanotecnologie, CNR, I-20133 Milan, Italy*

[4]*Department of Physics, TU Dortmund University, 44227 Dortmund, Germany*

[*]*corresponding authors*

Table of Contents

1. Theory of multi-mode coupling 2

2. Alternative visualisation of the data 3

3. FEFD simulations of light-matter coupling 6

4. Dependence of polariton formation as a function of the patch size 7

1. Theory of multi-mode coupling

In ref. [31] we developed a theory for the light-matter coupling between a given number of photonic resonator modes and the cyclotron resonances (CRs) of a 2DEG of carrier density ρ_{QW}. Whilst the full derivation of the multi-mode Hamiltonian is detailed in [31], we summarize the key points below.

The full vector potential of the electromagnetic field of the resonator can be expressed as a sum of the photonic modes with dimensionless spatial profile $f_\nu(\mathbf{r})$, frequency ω_ν, and photonic annihilation operator $\hat{a}_\nu$, as

$$\hat{A}(\boldsymbol{r}) = \sum_\nu \sqrt{\frac{\hbar}{2\,\epsilon_0 \epsilon_r \omega_\nu V_\nu}} f_\nu(\mathbf{r})\,(\hat{a}_\nu + \hat{a}_\nu^\dagger), \tag{1}$$

where V_ν is the mode volume, and ϵ_r is the background dielectric constant in the QW plane. Placing a 2DEG below the resonator reduces the dimensionality of the system and breaks the orthogonality of the different photonic modes, displayed by the non-zero integral matrix element

$$F_{\nu,\mu} = \int_S f_\nu^*(z, r_{||})\, f_\mu(z, r_{||})\, dr_{||}, \tag{2}$$

where $r_{||}$ is the in-plane vector, z is the out-of-plane coordinate of the 2DEG, and S is the in-plane domain, i.e., the QW surface. By remapping the non-orthogonal photonic modes $f_\nu(z, r_{||})$ onto an arbitrary basis of orthogonal in-plane functions $\phi_\nu(z, r_{||})$ as

$$f_\nu(z, r_{||}) = \sum_\mu \alpha_{\nu,\mu}\, \phi_\nu(z, r_{||}), \tag{3}$$

and, substituting Eq. 3 into the expression of the vector potential, one obtains a Hopfield-like Hamiltonian, which includes cross-interaction terms

$$\hat{H}_{int} = \sum_\nu \sum_{\mu \le \nu} \hbar\, [(\Omega_{R,\nu,\mu} \hat{b}_\mu + \Omega_{R,\nu,\mu}^* \hat{b}_\mu^\dagger)(\hat{a}_\nu^\dagger + \hat{a}_\nu)], \tag{4}$$

where a set of collective bosonic matter operators $\hat{b}_\nu$ represents in-plane degenerate but orthogonal modes for the electronic excitations.

The cross-interaction between the νth photon mode and μ^{th} electronic mode is described by the vacuum Rabi energies $\Omega_{R,\nu,\mu} \propto \alpha_{\nu,\mu}$, which can be expressed in terms of an overlap parameter defined as $\eta_{\nu,\mu} = \frac{F_{\nu,\mu}}{\sqrt{F_{\nu,\nu} F_{\mu,\mu}}}$. The parameter $\eta_{\nu,\mu}$ attains a value of 1 when the field distribution of the two modes is identical within the domain S. This is the case in our structured sample where the domain S is restricted to the central region of the resonator, where both modes overlap and differ only by a normalising factor which is included in an effective mode length $\tilde{V}_\nu = \frac{V_\nu}{F_{\nu,\nu}}$ in z-direction.

In the case of two photonic modes, the coupling strengths are given in full as:

$$\Omega_{\mathrm{R},1,1} = \sqrt{\frac{\omega_{\mathrm{c}} n_{\mathrm{QW}} \rho_{\mathrm{QW}} e^2}{2m^* \epsilon_0 \epsilon_r \omega_1 \tilde{V}_1}}, \tag{5}$$

$$\Omega_{\mathrm{R},2,1} = \sqrt{\frac{\omega_{\mathrm{c}} n_{\mathrm{QW}} \rho_{\mathrm{QW}} e^2}{2m^* \epsilon_0 \epsilon_r \omega_2 \tilde{V}_2}} \eta_{2,1}, \tag{6}$$

$$\Omega_{\mathrm{R},2,2} = \sqrt{\frac{\omega_{\mathrm{c}} n_{\mathrm{QW}} \rho_{\mathrm{QW}} e^2}{2m^* \epsilon_0 \epsilon_r \omega_2 \tilde{V}_2}} \sqrt{1 - |\eta_{2,1}|^2}. \tag{7}$$

2. Alternative visualisation of the data

In addition to the color maps shown in Figs. 3 and 4 of the manuscript, we provide the same data as waterfall plots in Figs. S1 and S2. The curves are ordered by the value of the cyclotron resonance frequency, ν_{c}, in vertically ascending order.

Moreover, since the LP_1 resonance is difficult to track in the 2D colormap of Fig. 4a owing to the comparably low oscillator strength, we show the magnetic field dependence of the LP_1, normalized to the average transmission $\mathcal{T}_{avg}$ for each frequency ν, in Fig. S3.

For a quantitative comparison of the oscillator strengths of the polariton resonances, we extract the values of the absorption of several of the coupled modes. For the experimental data, the transmission of the LP_1 and UP_1 modes of the unstructured sample reaches $\mathcal{T}_{\mathrm{LP}_1} \approx 0.26$ at ν_{c} = 1.9 THz, and $\mathcal{T}_{\mathrm{UP}_1} \approx 0.32$, at ν_{c} = 0 THz. In comparison, the absorption of the polariton modes of the structured sample vary more strongly, but reach similar minimal values of $\mathcal{T}_{\mathrm{LP}_1} \approx 0.21$ at ν_{c} = 1.9 THz, and $\mathcal{T}_{\mathrm{S-mode}} \approx 0.24$, at ν_{c} = 0 THz. A similar result is obtained from the FEFD simulations with $\mathcal{T}_{\mathrm{LP}_1} \approx 0.22$ and $\mathcal{T}_{\mathrm{UP}_1} \approx 0.22$ for the unstructured sample and $\mathcal{T}_{\mathrm{LP}_1} \approx 0.21$ and $\mathcal{T}_{\mathrm{S-mode}} \approx 0.21$ for the structured one, obtained in each case for the same ν_{c} as for the corresponding experimental data.

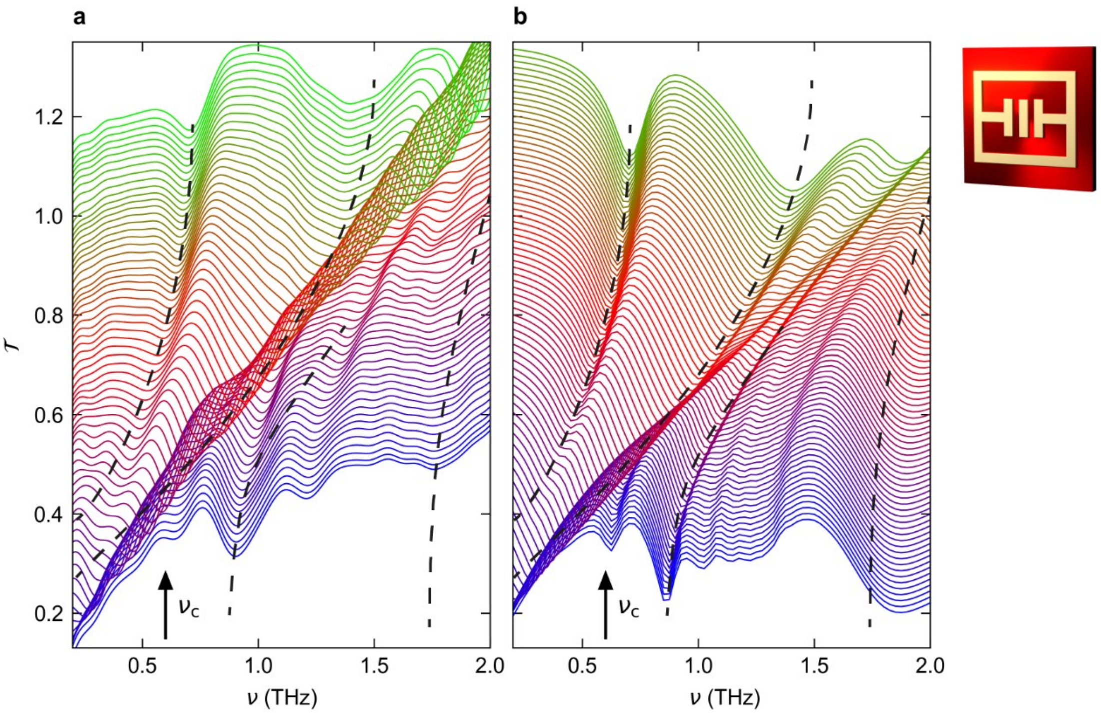

Figure S1: a, Experimental transmission spectra of the unstructured sample as a function of the cyclotron frequency, ν_c. The individual spectra are vertically offset for visual clarity. Dashed lines as a guide to the eye. **b**, Corresponding FEFD simulation of the transmission spectra. The individual spectra are vertically offset for visual clarity. Dashed lines as a guide to the eye. Outset: Schematic of the resonator on top of an unstructured QW (bright red area).

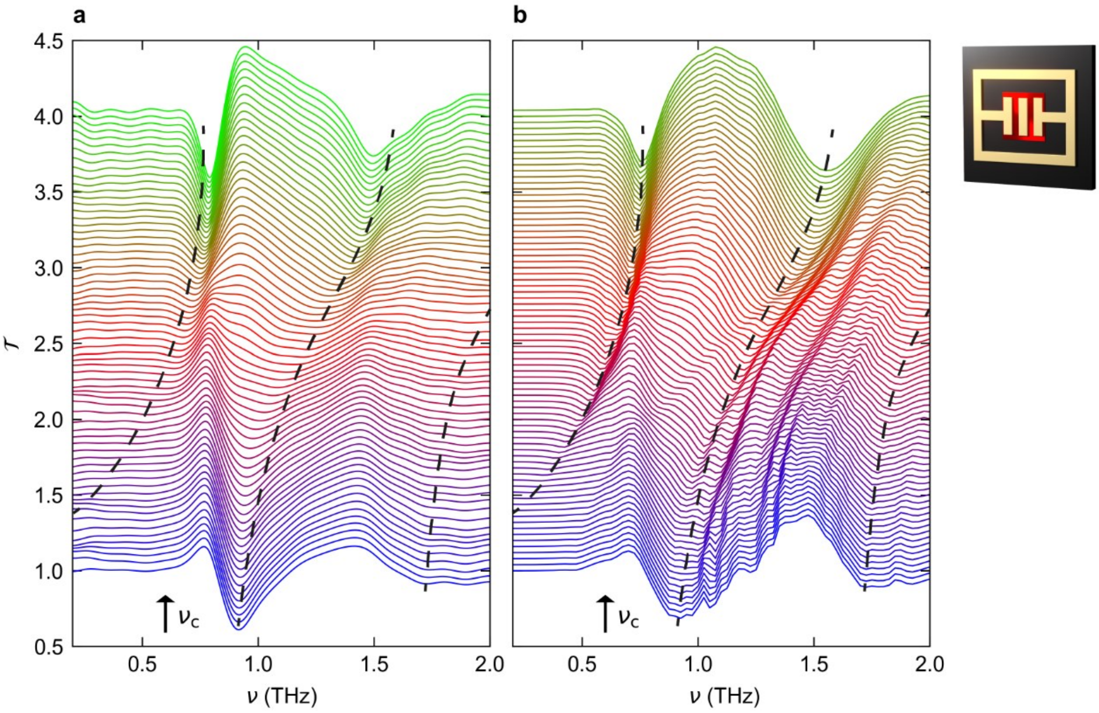

Figure S2: **a**, Experimental transmission spectra of the structured sample, each normalized to the average transmission $\mathcal{T}_{avg}$ for each frequency ν, as a function of the cyclotron frequency ν_c. The individual spectra are vertically offset for visual clarity. Dashed lines as a guide to the eye. **b**, Corresponding FEFD simulation of the transmission spectra. The individual spectra are vertically offset for visual clarity. Dashed lines as a guide to the eye. Outset: Schematic of the resonator on top of a structured QW (bright red area).

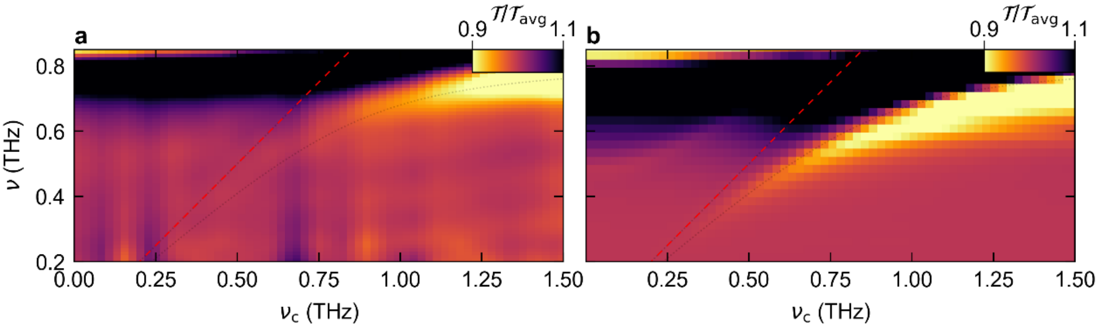

Figure S3: **a**, Experimental transmission spectra of the unstructured sample, each normalized to the average transmission $\mathcal{T}_{avg}$ for each frequency ν, as a function of the cyclotron frequency ν_c. The semi-transparent curves represent the eigenmodes calculated by our theoretical model. **b**, FEFD simulation of the transmission spectra, each normalized to the average transmission $\mathcal{T}_{avg}$ for each frequency ν, as a function of the cyclotron frequency ν_c, including the theoretical eigenmodes of panel **a**.

3. FEFD simulations of light-matter coupling

We perform finite-element frequency-domain (FEFD) simulations of the transmission of our coupled structures which solve Maxwell's equations on a discretized version of the sample geometry consisting of the GaAs substrate, the GaAs QW stack, the gold resonator structure, and vacuum. Details of this approach are given in Ref. [17]. We implement the QW response by a gyrotropic dielectric tensor parameterized by the cyclotron resonance frequency and an oscillator strength proportional to the charge carrier density. For the latter, we chose a value of $\rho_{\mathrm{QW}} = 1.25 \times 10^{12}\ \mathrm{cm}^{-2}$ for best agreement with the experiment, which is in close vicinity of the nominal chemical doping density of $1 \times 10^{12}\ \mathrm{cm}^{-2}$.

In an additional set of simulations, we investigate the role of higher-order modes. Generally, in a multi-mode light-matter coupled structure, modes within a spectral range comparable to or smaller than their vacuum Rabi frequencies, Ω_{R}, need to be considered. FEFD simulations for a frequency range of up to ν = 6 THz and up to ν_{c} = 6 THz (Fig. S4) show that higher-order photonic modes n ≥ 3 exhibit almost no light-matter coupling signatures and thus contribute only negligibly to the coupling mechanism in our structures. We have thus restricted the analysis to the LC and DP modes.

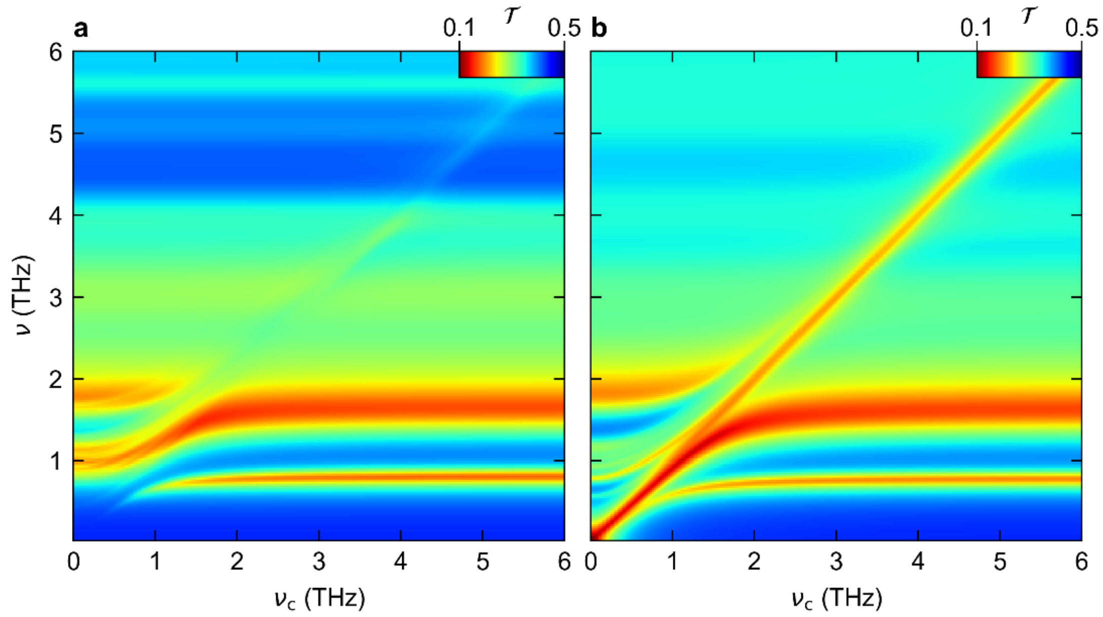

Figure S4: Wide-band FEFD simulation of the transmission spectra of **a**, the structured and **b**, the unstructured sample, as a function of the cyclotron resonance frequency, ν_{c}.

4. Dependence of polariton formation as a function of the patch size

In addition to the simulations supporting the two experimentally investigated cases, we provide additional simulations and fits based on our Hamiltonian model (Fig. S5), which systematically show the transition of the relevant coupling parameters between the two extremal cases. The numerical values are given in Table S1. The data show a clear, monotonic progression of the frequencies of the coupled modes as well as the vacuum Rabi frequencies, from the case of the smallest investigated patch size of 15 µm side length to the full, unstructured QW film. Moreover, the merging of the LP_2 and UP_1 resonances into the S-shaped mode is traced.

Finally, Fig. S6 shows the overlap parameter $\eta_{2,1}$ as a function of the patch size. The data indicate a comparably sharp transition from full overlap, $\eta_{2,1}$ = 1, to the limiting value of $\eta_{2,1}$= 0.15 for an unstructured, infinitely extended QW system. The experimentally investigated structures thus represent the extremes of both scenarios.

**Patch side length [µm]**	**15**	**20**	**25**	**30**	**35**	**Full QW**
$\frac{\Omega_{R,1,1}}{\omega_1}$	0.28	0.37	0.45	0.43	0.37	0.37
$\frac{\Omega_{R,2,1}}{\omega_1}$	0.27	0.31	0.38	0.27	0.26	0.07
$\frac{\Omega_{R,2,2}}{\omega_2}$	0.006	0.005	0.01	0.03	0.08	0.21
$\eta_{2,1}$	0.999	0.9995	0.9975	0.97	0.84	0.15

Table S1: Coupling parameters for the simulations with varying QW patch size.

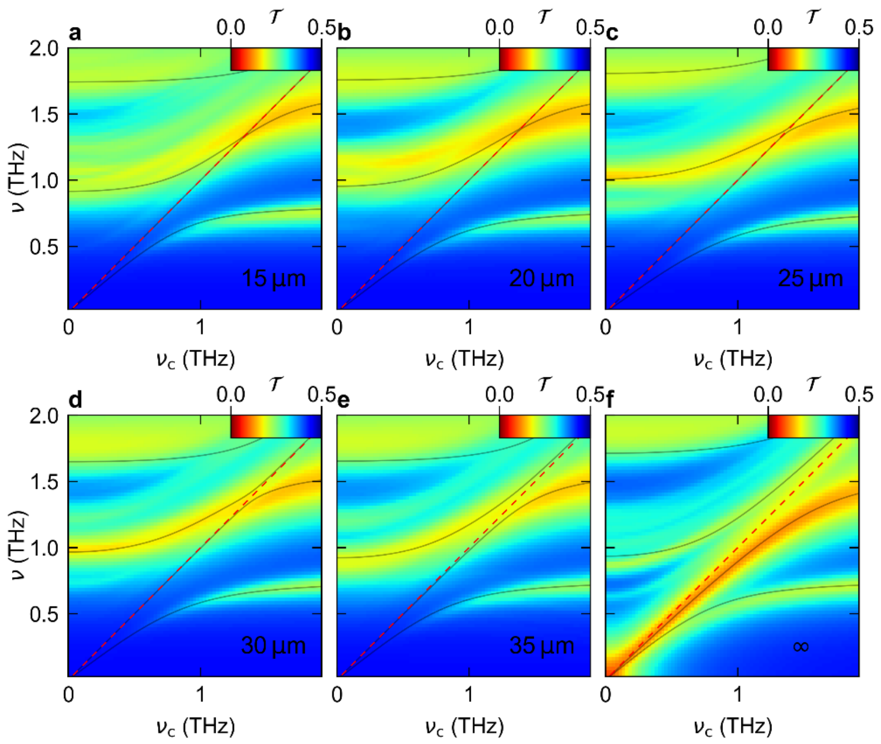

Figure S5: FEFD simulation of the transmission spectra of the structures as a function of the cyclotron frequency ν_c with different patch sizes and including individual fits with the multi-mode Hamiltonian. **a**, structured QW with a side length of 15 µm, **b**, 20 µm, **c**, 25 µm, **d**, 30 µm, **e**, 35 µm, and **f**, unstructured QW film.

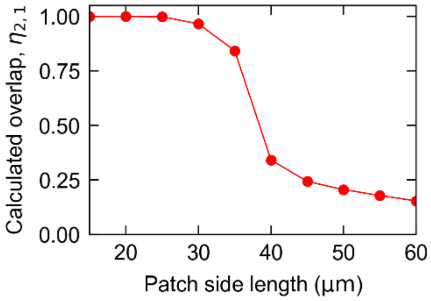

Figure S6: Overlap parameter $\eta_{2,1}$ as a function of the side length of the quadratic QW patches. The data are calculated from the simulated near-field distribution of the resonator modes.
